# Economic Burden Associated with Negative Symptoms Identified Through Natural Language Processing Among Patients with Schizophrenia in the United States

**DOI:** 10.1093/schbul/sbaf073

**Published:** 2025-06-03

**Authors:** Jerome Vaccaro, Mona Nili, Pin Xiang, James K Nelson, Cory Pack, Randall Thompson, Joe Vasey, Joseph Parks

**Affiliations:** RightPath HC, Millerton, NY 12546, United States; Boehringer Ingelheim Pharmaceutical Inc, Ridgefield, CT 06877, United States; Boehringer Ingelheim Pharmaceutical Inc, Ridgefield, CT 06877, United States; Veradigm Health, Raleigh, NC 27609, United States; Veradigm Health, Raleigh, NC 27609, United States; Veradigm Health, Raleigh, NC 27609, United States; Veradigm Health, Raleigh, NC 27609, United States; National Council for Behavioral Wellbeing, Washington, DC 20005, United States

**Keywords:** schizophrenia, negative symptoms, burden of illness, hospitalization

## Abstract

**Background and Hypothesis:**

The objective of this study was to identify documented negative symptoms of schizophrenia using natural language processing (NLP) and to characterize treatment patterns, healthcare resource utilization (HCRU), and costs among this patient population.

**Study Design:**

This US retrospective cohort study used electronic health records (EHR) linked to administrative claims data from January 2016 through February 2023. Adult patients (≥ 18 years) with at least 2 schizophrenia diagnosis codes were included. Negative symptoms were identified by NLP. Patient characteristics were assessed in the 12 months preceding the index date (first-documented schizophrenia diagnosis). Treatment patterns, HCRU, and costs were measured over the 12 months after the index date.

**Study Results:**

A total of 79,326 patients were enrolled in the EHR cohort and 14,992 (18.9%) had documented negative symptoms. Avolition was identified most often (44%) followed by blunted affect (42%). In the EHR cohort, 11,293 patients (14.2%) had linked claims. Patients with documented negative symptoms had more HCRU including days hospitalized, outpatient visits, and all-cause healthcare claims than patients without documented negative symptoms (all *P* < .001). They also had higher healthcare costs including inpatient, all-cause healthcare, schizophrenia-related costs (*P* < .001), and outpatient costs (*P* = .029). Only 34.6% of patients with documented negative symptoms received psychosocial interventions.

**Conclusions:**

These observational data add to the limited published literature on negative symptoms in patients with schizophrenia in the United States. The association of negative symptoms with low utilization of psychosocial interventions, increased costs, and high healthcare utilization emphasize the need for better management and treatment options for patients with schizophrenia.

## Introduction

Schizophrenia is a chronic and complex mental health condition affecting approximately 1.3-2.8 million adults in the United States, or 0.5%-1% of the population.^1–3^ Once diagnosed, patients have a poor prognosis, with less than 14% achieving a sustained recovery, which is defined as both symptomatic remission (at most, mild symptoms) and improved social functioning for more than 2 years.^4^ Costs associated with schizophrenia lead to a substantial economic burden, with the total annual per patient direct and indirect costs estimated at over $44,000.^5^ The resulting total direct and indirect costs of schizophrenia in the United States are estimated to be over $155 billion per year.^5^ These costs are nearly 2.5 times greater than the estimated total costs in 2002 of 62.7 billion per year.^6^

Schizophrenia is characterized by a mix of positive and negative symptoms as well as cognitive impairments.^7–9^ Positive symptoms are those that are noted by their “presence,” and include reality distortion symptoms such as hallucinations, delusions, disorganized movement, and thought disorders such as disorganized speech.^7–9^ Cognitive impairments associated with schizophrenia are expressed through attention and concentration deficits, memory dysfunction, and aspects of executive functioning.^10–12^

Negative symptoms refer to the absence of or a reduction in healthy emotions and behaviors and are an important identifying component of schizophrenia.^13^ Negative symptoms consist of 5 domains that can be grouped into the experiential dimension (avolition, asociality, and anhedonia) and the expressive dimension (alogia, blunted affect). These symptoms are typically long-lasting, persist throughout the course of schizophrenia, and tend to become more prominent over time.^13,14^ Patients can lack self-awareness of these symptoms as part of their illness. Clinicians have more training in assessing and treating positive symptoms, making the measurement of negative symptoms in clinical practice challenging and uncommon.^13,15^ Clinical recognition is important because negative symptoms substantially impact functioning and activities of daily living across the life span of people with schizophrenia.^10,16^ Negative symptoms are associated with several comorbidities including dyslipidemia, arterial hypertension, obesity, and diabetes mellitus, and can have an adverse impact on treatment adherence.^17–19^

Patients living with schizophrenia may experience symptoms from multiple domains, but FDA-approved pharmacological treatment options only affect the expression of positive symptoms.^20^ Currently, there are no FDA-approved pharmacological treatments indicated for patients with negative symptoms.^20^ Negative symptoms may still occur and develop when a pharmacological treatment, such as an antipsychotic, is used to treat positive symptoms.^21–23^ In fact, some medications are thought to worsen negative symptoms.^23,24^

Structured evaluation of negative symptoms in regular charting is not routinely done in clinical practice, making it unclear what the true burden of negative symptoms is for patients living with schizophrenia. The added functional and clinical burden of negative symptoms combined with a lack of access to effective treatments may result in negative symptoms as a contributing source to the increasing economic burden of schizophrenia. Real-world evidence studies focusing on negative symptoms and their economic impact are limited.^25,26^

Natural language processing (NLP) is a tool that can aid in better identification of negative symptoms and evaluation of their impact on patients with schizophrenia. Natural language processing is a technology that allows computers to process and analyze large amounts of natural language data as well as identify clinical data recorded in the free-text narratives of electronic health records (EHR). It provides a method for ascertaining detailed descriptive patient information from large volumes of clinical data.

Accordingly, the objectives of this study were 2-fold. First, we aimed to use EHR data to identify documented negative symptoms among patients with schizophrenia using NLP and to describe the demographic and clinical characteristics of these patients. Second, we sought to link EHR data to insurance claims to characterize treatment patterns, healthcare resource utilization, and costs among patients with schizophrenia with or without negative symptoms.

## Methods

### Study Design

This retrospective cohort study used existing EHR data from January 01, 2016, through February 28, 2023, to accomplish both objectives of the study. In addition, in furtherance of the second objective, the EHR data were linked to administrative insurance claims to create the linked claims cohort.

### Study Population

The study included adult patients (≥ 18 years) with a diagnosis of schizophrenia (ICD-9: 295.XX; ICD-10-CM: F20.X) at any time between January 1, 2016 and February 28, 2023. The index date was defined as the date of the first schizophrenia diagnosis within this time period. Participants also must have experienced 2 or more schizophrenia outpatient encounters on or after January 1, 2016, and at least 12 months of EHR activity prior to the first schizophrenia diagnosis (EHR cohort). Patients with a diagnosis of dementia or cognitive impairments (dementia, frontotemporal lobe disease, prion disease, autism spectrum disorder, epilepsy, intellectual disability, multiple sclerosis, Parkinson’s disease, stroke, or traumatic brain injury) at any time were excluded. For the second objective, a subgroup analysis was conducted with EHR data linked to administrative claims. This linked claims cohort only included patients who were continuously enrolled in medical and pharmacy benefits (i.e., with available claims data) for ≥ 1 year after the index date (linked claims cohort).

### Data Sources

Data was sourced from the Practice Fusion (PF) and Next Gen EHR databases, components of the Veradigm Health Insights ambulatory (outpatient) medical facilities throughout the United States. The practice sizes in the data were small (1-5 providers) to medium (< 20 providers) and most prevalently located in the South, Southwest, and Northeast of the United States. The PF dataset included available, unstructured clinical notes for searching and NLP. These notes potentially captured signs, symptoms, observations, and assessments that could identify negative symptoms not typically available in structured EHR data.

In order to create the linked claims cohort for purposes of the second study objective, the PF-EHR data were linked to closed (fully adjudicated, full enrollment) claims data. De-identified EHR and claims records were linked using Datavant’s deterministic matching solution (Datavant is a data logistics company for health care (Datavant.com)). This solution utilized encrypted protected health information derived from tokens in both data sources to facilitate patient linking of de-identified data. Claims records included procedural, medical, and pharmaceutical claims from approximately 150 US health insurance vendors. The linked data set contained the all-cause healthcare information of patients across the United States who had claims in the claims data set and who had interacted with a healthcare provider using the PF-EHR system (linked claims cohort).

Anderson’s behavioral health model guided the selection of demographic and clinical variables associated with healthcare resource utilization.^27^ Andersen’s framework was used for this study for several reasons: (1) it enabled adjustment for a comprehensive list of factors associated with healthcare utilization; (2) it is well-suited for studies that have many variables representing the same domain; and (3) it is extensively used in health services research to explain healthcare utilization. Sociodemographic variables for both the EHR cohort and the linked claims cohort included age, sex, race, ethnicity, census region, and payer type. Clinical variables were the Charlson Comorbidity Index (CCI) score and comorbid conditions,^28^ body mass index (BMI), and psychiatric comorbidities. The CCI score, a weighted composite score, predicts 1-year mortality based on the presence of select comorbidities. A high score indicates a higher risk of mortality. Sociodemographic characteristics were assessed at the index date. CCI score and comorbid component conditions were assessed over the 12-month pre-index period. The BMI value used was the closest recorded value to the index date within 12 months.

The treatment patterns, all-cause healthcare resource utilization, and costs were measured over the 12 months after the index date for the linked claims cohort. The treatment pattern variables were pharmacologic therapy (i.e., first- and second-generation antipsychotics, mood stabilizers, antidepressants) and psychosocial interventions (ie, cognitive behavioral therapy, psychotherapy, family therapy, psychosocial rehabilitation, and/or group therapy). The all-cause healthcare resource utilization variables were outpatient visits, inpatient admissions, duration of inpatient admissions, emergency department visits, pharmacy claims, and psychosocial service visits. The cost variables were inpatient and outpatient costs, all healthcare-related costs, and schizophrenia-related healthcare costs.

### Natural Language Processing (NLP) Analysis

Natural language processing was used to identify negative symptoms in the clinical notes for both the EHR cohort and the linked claims cohort. All unstructured texts from patient records during the study period were processed for review. The steps of NLP were:

- *Data Preprocessing.* In this initial step, Python scripts were used to remove hypertext markup language (HTML) tags from clinical notes, converting them into plain text. This process required writing code to identify and eliminate HTML-specific syntax, ensuring the retention of only the actual text content for subsequent analysis.
- *Text Segmentation.* Once the text was clean of HTML tags, NLP libraries in Python were used to segment the text into individual sentences. This sentence segmentation analyzed punctuation and syntax to accurately delineate the end of one sentence and the beginning of another.
- *Keyword Extraction:* This process began with the creation of an initial negative symptoms list by the research team, in collaboration with medical informatics specialists and clinical experts. The team used the accepted latent structure of negative symptoms as best conceptualized by 5 key domains: blunted (or flat) affect (reduced range of emotions), alogia (reduction in the number of words spoken), avolition or lack of volition (reduced goal-directed activity due to decreased motivation), asociality (social withdrawal), and anhedonia (reduced experience of pleasure).^29,30^ To ascertain synonyms and frequent misspellings associated with negative symptoms, consultations were held with the study team to create a list of key terms. This initial list served as a starting point for scanning the data. Utilizing this list, a comprehensive search of clinical notes in the dataset was conducted employing regular expressions, keyword searches, and Levenshtein distance algorithms to identify further variations, including synonyms, acronyms, abbreviations, and misspellings. This iterative and data-driven approach included a clinician’s review of intermediate results to refine the list systematically. The additional phrases were subsequently converted into regular expression patterns allowing for efficient and precise identification of specific keywords and phrases in the data. A set of regular expression patterns corresponding to relevant keywords was established (see Supplementary Table S1). Sentences meeting predefined criteria of negation, questions, goals, or references to family history were excluded from further analysis. These rules effectively filtered out sentences that did not qualify as presenting negative symptoms, ensuring the study focused on relevant data.
- *Quality Control:* A manual review of a randomly selected subset of the processed data (*n* = 250 patients) was conducted to ensure the reliability of the analysis. In this step, the outcomes of the Python NLP analysis were compared with manual interpretations to verify the accuracy and effectiveness of the algorithms used. F1 scores were calculated for each category of negative symptoms. An F1 score is an industry-standard machine-learning evaluation metric that measures a model’s performance.^31^ It combines the precision and recall scores of a model and computes how many times a model made a correct prediction across the entire dataset. The F1 score is calculated as the harmonic mean of the precision and recall scores with the equation: F1 = 2 * (precision * recall) / (precision + recall). The resulting score ranges from 0% to 100%, with a higher F1 score denoting a more accurate classifier. An F1 score of > 0.8 is considered a good performance. A confusion matrix was also produced to calculate other model performance metrics including true positives, true negatives, false positives, and false negatives (see Supplementary Table S2). The model precision and recall for the 5 key domains ranged from 0.96% to 100% (precision) and from 0.67% to 86% (recall).

### Statistical Analysis

Descriptive reporting was stratified by the presence or absence of negative symptoms in the EHR cohort and the linked claims cohort. Results were described as counts and percentages for categorical variables and measures of central tendency (mean, median, and standard deviation [SD]). Simple statistical comparisons between cohorts were conducted using chi-square and *t*-test.

This study used a two-part regression model for calculating the adjusted estimate of all-cause total costs. The first part of the two-part model was a logistic regression, and the second part was a generalized linear model (GLM) with gamma distribution and log link. In addition, this study used a negative binomial regression model for calculating the adjusted estimates of inpatient admission. All the sociodemographic (age, gender, race, and region) and clinical characteristics (CCI score, anxiety, bipolar disorder, depression, panic disorder, post-traumatic stress disorder, and substance use disorder) were included in the regression models for calculating adjusted estimates.

## Results

**Figure 1.**
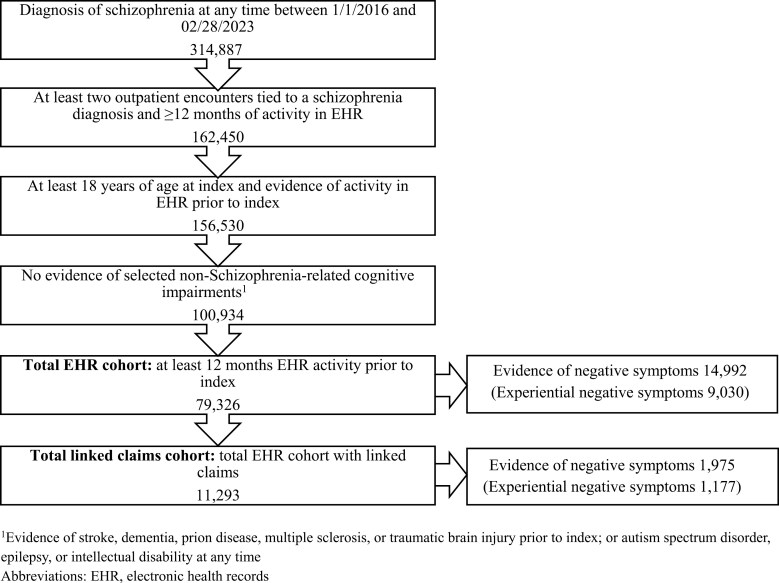
Study Population ^1^Evidence of stroke, dementia, prion disease, multiple sclerosis, or traumatic brain injury prior to index; or autism spectrum disorder, epilepsy, or intellectual disability at any time. Abbreviations: EHR, electronic health records

Between January 1, 2016 and February 28, 2023, 314,887 patients with schizophrenia were assessed for eligibility, of whom 79,326 (78.6%) met the selection criteria and were enrolled in the EHR cohort. Among these patients, 14,992 (18.9%) had documented evidence of negative symptoms identified by NLP. The majority of the negative symptoms documented in the clinical notes were in the experiential dimension (60.2%). Of the total patients in the EHR cohort, 11,293 (14.2%) had linked claims. Out of this linked-claims cohort, 1975 (17.5%) had negative symptoms. The final study cohorts are shown in Figure 1. Additional details on the selection criteria of the study population are provided in Supplementary Table S3.

Patients with and without documented negative symptoms in the EHR cohort and in the linked claims cohort were generally balanced in terms of gender, race, and BMI. In the EHR cohort, patients with negative symptoms tended to be younger compared to those with no negative symptoms (49.3 vs 51.0 years old, *P* < .001). Similarly, in the linked claims cohort, those with negative symptoms were younger than those without (46.2 vs 47.8 years old, *P* < .001). Patients with negative symptoms in the EHR cohort had fewer comorbidities compared to those without negative symptoms (CCI = 0.41 vs 0.47, *P* < .001). In the linked claims cohort, there were no differences in comorbidities between those with and without negative symptoms (CCI = 0.78 vs 0.80, *P* = .522).

In the EHR cohort, those with negative symptoms had a higher percentage of psychiatric conditions including anxiety (18.7% vs 15.7%), depression (19.0% vs 17.3%), post-traumatic stress disorder (5.9% vs 4.0%), and substance use disorder (17.7% vs 15.4%) compared to those with no negative symptoms (all comparisons *P* < .001). Similarly, in the linked claims cohort, those with negative symptoms tended to have a higher percentage of psychiatric conditions including anxiety (38.5% vs 29.4%), bipolar disorder (24.2% vs 21.3%), depression (36.4% vs 31.3%), panic disorder (4.1% vs 2.8%), post-traumatic stress disorder (11.4% vs 8.2%), and substance use disorder (34.4% vs 30.7%) compared to those with no negative symptoms (all comparisons *P* < .01). Table 1 shows the sociodemographic and clinical characteristics for study patients in the EHR cohort and in the linked claims cohort.

**Table 1.** Patient Sociodemographic and Clinical Characteristics

	EHR cohort					Linked claims cohort				
Variable	With documented negative symptoms		No documented negative symptoms		*P* value	With documented negative symptoms		No documented negative symptoms		*P* value
	*N* = 14,992		*N* = 64,334			*N* = 1,975		*N* = 9,318		
Age, years (mean, SD)	49.3	15.2	51.0	14.8	<.001	46.2	14.5	47.8	14.2	<.001
Age group (*N*, %)					<.001					<.001
18–44	5,945	39.7%	22,015	34.2%		927	46.9%	3,885	41.7%	
45–64	6,439	42.9%	30,193	46.9%		840	42.5%	4,409	47.3%	
65+	2,608	17.4%	12,126	18.8%		208	10.5%	1,024	11.0%	
Gender (*N*, %)					.035					.080
Male	9,298	62.0%	39,171	60.9%		1,271	64.4%	5,801	62.3%	
Female	5,689	37.9%	25,136	39.1%		704	35.6%	3,517	37.7%	
Race (*N*, %)					<.001					.002
White	6,793	45.3%	28,070	43.6%		846	42.8%	3,904	41.9%	
Black	3,355	22.4%	15,875	24.7%		468	23.7%	2,483	26.6%	
Asian	757	5.0%	3,371	5.2%		101	5.1%	519	5.6%	
Other	2,159	14.4%	10,105	15.7%		344	17.4%	1,620	17.4%	
Unknown/Not reported	1,928	12.9%	6,913	10.7%		216	10.9%	792	8.5%	
Ethnicity (*N*, %)					<.001					<.001
Hispanic	739	4.9%	2,932	4.6%		149	7.5%	540	5.8%	
Non-Hispanic	10,462	69.8%	31,780	49.4%		1,366	69.2%	4,954	53.2%	
Unknown/Not Reported	3,791	25.3%	29,622	46.0%		460	23.3%	3,824	41.0%	
Region (*N*, %)					<.001					.001
Northeast	1,745	11.6%	7,890	12.3%		290	14.7%	1,189	12.8%	
Midwest	3,307	22.1%	12,510	19.4%		310	15.7%	1,515	16.3%	
South	4,407	29.4%	17,925	27.9%		546	27.6%	2,270	24.4%	
West	4,871	32.5%	22,662	35.2%		790	40.0%	4,151	44.5%	
Other/Unknown	662	4.4%	3,347	5.2%		39	2.0%	193	2.1%	
BMI (mean, SD)	30.2	6.4	30.2	6.3	.101	29.9	6.5	30.0	6.3	.267
CCI (mean, SD)	0.41	1.01	0.47	1.08	<.001	0.78	1.36	0.80	1.45	.522
CCI conditions^1^ (*N*, %)										
Any malignancy	185	1.2%	1,118	1.7%	<.001	49	2.5%	257	2.8%	.491
Cerebrovascular disease	–	<1%	–	<1%	–	27	1.4%	157	1.7%	.311
Chronic pulmonary disease	1,769	11.8%	8,386	13.0%	<.001	409	20.7%	2,044	21.9%	.230
Congestive heart failure	327	2.2%	1,813	2.8%	<.001	76	3.8%	435	4.7%	.111
Diabetes, chronic complications	725	4.8%	3,676	5.7%	<.001	187	9.5%	833	8.9%	.457
Diabetes without complications	2,115	14.1%	10,714	16.7%	<.001	458	23.2%	2,148	23.1%	.895
Liver disease, mild	541	3.6%	2,539	3.9%	.054	162	8.2%	743	8.0%	.734
Myocardial infarction	–	<1%	–	<1%	–	30	1.5%	186	2.0%	.160
Peripheral vascular disease	569	3.8%	2,347	3.6%	.388	142	7.2%	593	6.4%	.177
Renal disease	472	3.1%	2,696	4.2%	<.001	104	5.3%	520	5.6%	.578
Rheumatic disease	–	<1%	–	<1%	–	32	1.6%	146	1.6%	.863
Hypertension	3,459	23.1%	18,031	28.0%	<.001	803	40.7%	3,894	41.8%	.354
Psychiatric conditions (*N*, %)										
Anxiety	2,800	18.7%	10,075	15.7%	<.001	760	38.5%	2,742	29.4%	<.001
Bipolar disorder	1,774	11.8%	6,848	10.6%	<.001	477	24.2%	1,989	21.3%	.006
Depression	2,847	19.0%	11,151	17.3%	<.001	719	36.4%	2,913	31.3%	<.001
Panic disorder	263	1.8%	789	1.2%	<.001	81	4.1%	260	2.8%	.002
Post-traumatic stress disorder	884	5.9%	2,605	4.0%	<.001	225	11.4%	765	8.2%	<.001
Substance use disorder	2,654	17.7%	9,936	15.4%	<.001	679	34.4%	2,863	30.7%	.002
Payer (*N*, %)					<.001					.045
Commercial	296	2.0%	1,002	1.6%		101	5.1%	434	4.7%	
Dual Medicare-Medicaid	318	2.1%	1,332	2.1%		167	8.5%	792	8.5%	
Medicaid	2,448	16.3%	10,357	16.1%		1,420	71.9%	6,560	70.4%	
Medicare	278	1.9%	1,471	2.3%		121	6.1%	732	7.9%	
Self-insured	29	0.2%	100	0.2%		12	0.6%	40	0.4%	
Other	47	0.3%	281	0.4%		10	0.5%	91	1.0%	
Unknown	11,576	77.2%	49,791	77.4%		144	7.3%	669	7.2%	

^1^Only CCI conditions prevalent in ≥ 1% were included in the table. Abbreviations: BMI, body mass index; CCI, Charlson Comorbidity Index; EHR, electronic health record.

### Identification of Negative Symptoms in the EHR Cohort

The F1 score of the NLP model achieved more than 80% for all 5 concepts of negative symptoms indicating good performance of the developed model. The dimensions of negative symptoms identified most frequently in the EHR cohort were avolition or lack of volition (44%); blunted affect (42%); alogia (25.0%); anhedonia (22.0%); and asociality (5.4%) (Table 2).

**Table 2.** Five Dimensions of Negative Symptoms and the Most Commonly Occurring Terms or Phrases Associated with Them Found in Clinical Notes of the EHR Cohort^*^

Negative symptoms of schizophrenia within each main category^1^	Patients identified with each type of negative symptom out of the total number of patients with evidence of any negative symptoms in the EHR cohort, *N* = 14,992 (18.9%)
	*N* (%)
1.Blunted (or flat) affect (reduced range of emotions)	6,277 (41.9%)
Flat affect	4,608 (30.7%)
Poor eye contact	2,208 (14.7%)
2.Avolition (reduced goal-directed activity due to decreased motivation)	6,592 (44.0%)
Withdrawn	3,376 (22.6%)
Poor hygiene	2,670 (17.9%)
No motivation	711 (4.8%)
Poor grooming	307 (2.1%)
Loss of interest in activities	194 (1.3%)
Passivity	84 (0.6%)
3.Alogia (reduction in quantity of words spoken)	3,744 (25.0%)
No response	2,332 (15.6%)
Poverty of speech	844 (5.7%)
Short answers	305 (2.0%)
Not spontaneous	132 (0.9%)
Brief answers	150 (1.0%)
Minimal response	94 (0.6%)
4.Asociality (lack of motivation to engage in social interaction)	812 (5.4%)
Social withdrawal	501 (3.4%)
No friends	138 (0.9%)
Few friends	104 (0.7%)
5.Anhedonia (reduced experience of pleasure)	3,303 (22.0%)
Loss of interest	1,587 (10.6%)
Apathetic	1,286 (13.2%)
Apathy	753 (7.5%)

^*^NLP analysis was performed on the EHR cohort only.

^1^Negative symptoms identified by NLP are not mutually exclusive. Patients may have experienced multiple terms listed in the healthcare provider clinical notes.

Within the 5 overall concepts, the most common terms or phrases identified included “flat affect” (31%) and “poor eye contact” (15%) for blunted affect; “withdrawn” (23%) and “poor hygiene” (18%) for avolition; “no response” (16%) and “poverty of speech” (6%) for alogia; “loss of interest” (11%) and “apathetic” (13%) for anhedonia; and “social withdrawal” (3.4%) for asociality.

The number of clinical notes was associated with a higher proportion of patients with documented negative symptoms. In patients with > 50 clinical notes, 41.5% had documented negative symptoms; in patients with ≤ 5 clinical notes, 1.5% had documented negative symptoms.

### Healthcare Resource Utilization in the Linked Claims Cohort

Table 3 presents the annual all-cause healthcare resource utilization for schizophrenia patients in the linked claims cohort with and without documented evidence of negative symptoms. Study patients with negative symptoms documented in the clinical notes had more outpatient visits compared to those without documented evidence of negative symptoms (46.9 visits vs 40.2 visits, *P* < .001). Similarly, the patients with negative symptoms had a greater annual number of days hospitalized compared to the no-negative-symptoms cohort (5.2 vs 3.9 days, *P* < .001). Those with negative symptoms had a higher number of all-cause healthcare claims (including emergency department, inpatient, outpatient, and pharmacy) compared to those patients without evidence of negative symptoms (86.4 vs 78.0 claims, *P* < .001). Over 80% of patients in both groups utilized antipsychotic medications. A significantly higher percentage of individuals with negative symptoms were receiving psychosocial interventions (34.6% vs 28.3%, *P* < .001). However, among these patients, the average per patient per year (PPPY) number of psychosocial service visits did not significantly differ between patients with negative symptoms and those without negative symptoms (8.9 vs 9.3, *P* = .390) (median 3.7 for patients with and 3.1 for patients without negative symptoms).

**Table 3.** Annual Healthcare Resource Utilization for Patients in the Linked Claims Cohort

	With documented negative symptoms	No documented negative symptoms	*P* value
	*N* = 1,975	*N* = 9,318	
Annual Healthcare Resource Utilization PPPY (Mean)			
Number of outpatient visits	46.9	40.2	<.001
Total hospitalized days	5.2	3.9	<.001
Number of hospitalizations	0.4	0.3	.001
Number of emergency department visits	1.6	1.3	.112
Number of all-cause claims^1^	86.4	78.0	<.001
Number of pharmacy claims	37.2	36.0	.137
Receiving psychosocial interventions, %	34.6%	28.3%	<.001
Cognitive behavioral therapy %	0.2%	0.1%	.061
Psychotherapy %	31.6%	24.8%	<.001
Family therapy %	0.6%	0.8%	.386
Psychosocial rehabilitation %	3.6%	4.2%	.221
Group therapy/Counseling	4.3%	2.9%	<.001
Number of psychosocial service visits	8.9	9.3	.390
Any psychiatric pharmacy utilization, %	86.2%	83.7%	<.001
First-generation antipsychotics	20.4%	19.2%	.225
Second-generation antipsychotics	76.9%	72.1%	<.001
Mood stabilizers	22.1%	18.7%	<.001
Antidepressants	54.3%	51.8%	.045

^1^Overall healthcare claims include emergency department, inpatient, outpatient, and pharmacy.

### Healthcare Costs in the Linked Claims Cohort

**Figure 2.**
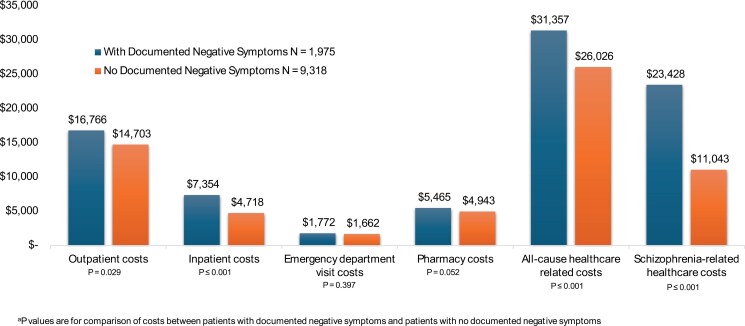
Annual Healthcare Costs for Patients in the Linked Claims Cohort

Patients with evidence of negative symptoms had significantly higher annual healthcare costs across several metrics compared to those study patients without negative symptoms (Figure 2). Patients with negative symptoms had nearly 60% higher mean PPPY inpatient costs ($7354.2 vs $4717.6, *P* < .001). The PPPY outpatient costs were also higher for those with negative symptoms ($16,765.6 vs $14,703.4, *P* = .029). The mean PPPY all-cause healthcare costs were almost 20% higher for those with documented negative symptoms ($31,356.5 vs $26,026.0, *P* < .001). The PPPY schizophrenia-related healthcare costs were almost twice as much for the patients with negative symptoms compared to those with no evidence of negative symptoms ($23,428.4 vs $11,042.6, *P* < .001).

The adjusted estimates for PPPY all-cause cost for those with the evidence of negative symptoms was $5896 higher compared to those without negative symptoms ($32,187.00 vs $26,291.04, *P* < .001). The adjusted estimates for inpatient admission PPPY for those with documented NS were 1.3 times higher than for those without documented NS (0.4 vs 0.3, *P* < .001). All *P* values were less than .001.

The results for patients with documented experiential negative symptoms were consistent with the overall results in those with negative symptoms (see Supplementary Tables S4 and S5).

## Discussion

In this large EHR cohort of schizophrenic patients, using NLP, we found an overall lower proportion of patients with documented negative symptoms compared to previous findings indicating approximately 60% of schizophrenia patients experienced negative symptoms. In our study, 18.9% of patients in the EHR cohort and 17.5% of patients in the linked claims cohort had documented negative symptoms in their clinical notes. In comparison, Bobes et al. (2010) found that one or more negative symptoms were present in 57.6% of 1704 stable adult psychiatric outpatients with schizophrenia.^32^ A similarly high prevalence was reported in a review of 20 placebo-controlled studies of second-generation antipsychotics with a combined sample of *n* = 7450, where 62% of patients had prominent negative symptoms.^33^ The American Psychiatric Association states that a mental status examination should be an integral part of every initial assessment.^34^ However, guidelines do not recommend a specific scale.^15,34^ Furthermore, negative symptoms are complex and varied and may be difficult to differentiate from conditions with overlapping symptoms such as depression, medication side effects, substance use, or neurological conditions.^8,10^

Our study adds to the limited real-world evidence studies conducted in the US focusing on negative symptoms and the potential economic impact.^25,26^ When we linked the EHR cohort data to administrative insurance claims, we created a linked claims cohort. We found a positive association between patients with schizophrenia with documented observed negative symptoms and a greater number of annual outpatient visits (46.9 vs 40.2, *P* < .001), more annual hospitalized days (5.2 vs 3.9, *P* < .001), and greater number of all-cause healthcare claims (86.4 vs 78.0, *P* < .001) compared to study participants without negative symptoms. This finding of increased healthcare utilization in patients with negative symptoms was also seen by Lavalee et al. (2019). In their US real-world database study, 8402 patients with negative symptoms had a significantly greater number of outpatient visits (8.4 vs 5.5, *P* < .001), inpatient admissions (6.8 vs 4.5, *P* < .001), longer inpatient stays (74.6 days vs 66.3 days, *P* = .001), and more psychotherapy visits (5 vs 4, P = .04) compared to the same number of patients without negative symptoms.^25^ A more recent longitudinal study utilizing de-identified administrative claims data from 2016 to 2022 had similar results. After controlling for baseline characteristics, patients with evidence of diagnosed negative symptoms (*N* = 5691) had greater annual all-cause inpatient admissions (mean: 5.2 vs 4.2), outpatient emergency room (ER) visits (mean: 2.8 vs 2.0), inpatient stay costs ($23,830 vs $20,669), outpatient ER visit costs ($1,738 vs $1,167), and lower prescription numbers (mean: 49.2 vs 51.4) compared to patients without negative symptoms (*N* = 236,895).^26^

While individuals in our study with negative symptoms received psychosocial interventions at a significantly higher percentage than those without negative symptoms (34.6% vs 28.3%, *P* < .001), this finding indicated that approximately two-thirds of patients were not utilizing psychosocial resources. Among the approximately one-third of patients who received psychosocial interventions, the average number of psychosocial service visits over one year did not significantly differ between patients with negative symptoms (8.9) and those without (9.3). The median number of days annually with a psychosocial service visit was 3.7 for those with negative symptoms and 3.1 for those without. This indicated that even for the one-third of patients receiving these resources, one-half of these patients had a visit less than 3.7 or 3.1 times per year.

Guidelines from the American Psychiatric Association suggest that patients with schizophrenia be treated with supportive psychotherapy and receive psychoeducation.^34^ Specific recommended psychosocial interventions also include cognitive behavioral therapy, cognitive remediation therapy, and social skills training. Access to mental health care in general is insufficient in the United States despite high demand.^35^ From national survey data published in 2022, almost a quarter of all adults with any mental illness reported that they were not able to receive the mental health services they needed.^35^ Reported barriers to getting that help included affordability, which encompassed lack of or limited insurance coverage, and the further complication that many providers did not take insurance. Other barriers included a lack of available treatment types, a shortage of providers, and a disconnect between primary care systems and behavioral health systems.

Previous studies also reported low rates of psychological therapy for schizophrenia.^36,37^ Coleman et al. (2016), who used EHR and linked claims data from 11 private, not-for-profit healthcare organizations (with a combined 7,523,956 adult patients receiving care in 2011), found a low rate of psychotherapy utilization among patients with schizophrenia.^36^ Of the 1,169,993 (15.6%) patients with a mental health diagnosis during that year, 15,105 had schizophrenia and less than 1% of these patients received psychotherapy. In comparison, 11% and 15% of patients with a diagnosis of anxiety disorder or depressive disorder received psychotherapy, respectively, demonstrating differences in the treatment of mental health conditions in the United States. Reist et al. (2021) found a low utilization of psychotherapy with first-episode psychosis in Missouri Medicaid claims data.^37^ Individuals with first-episode psychosis were identified (*N* = 6246 participants), and follow-up lasted a mean of 4.24 years. In this sample, 71% of patients with schizophrenia experiencing a first-episode psychosis had no evidence of receiving psychotherapy. Low utilization of psychosocial interventions in patients with schizophrenia indicated the need for a novel treatment modality to ensure access and equity for this underserved population.

There are limited previous studies on the impact of negative symptoms on patients with schizophrenia in the United States. Our study demonstrates the successful use of NLP in EHR to identify negative symptoms and offers new insights into the characteristics of negative symptoms in schizophrenia and the resulting economic impact. The results of this study highlight the value of advanced data analytics of real-world data in psychiatric research to provide a more comprehensive understanding of the patient experience. A further strength of this study is that it utilized a large sample size with over 79,000 schizophrenia patients in the total EHR cohort and over 11,000 patients in the linked claims cohort.

Some important limitations should be considered. Although this approach of using NLP facilitated an analysis that concentrated on text segments most relevant to identifying negative symptoms in the clinical notes, it is possible and even likely that this process underestimated the true prevalence of this important symptom domain for patients with schizophrenia. The low identification of documented negative symptoms in the clinical notes identified using NLP did not necessarily indicate the absence of negative symptoms. It is possible that clinicians gave less regard to negative symptoms when not using structured assessments. This study did show more documented negative symptoms in patients with a greater number of clinical notes. This possibly indicates that providers who made more clinical notes were more inclined to document negative symptoms. It is also possible that this association between negative symptoms and greater healthcare utilization could be caused by patients with more healthcare visits having a greater likelihood of having clinical notes recorded but we could not determine causality in this observational study. Inherent in all observational research is the inability to infer causality. Instead, with this observational study design we intended to raise the question of how frequently negative symptoms are documented for these patients and to describe characteristics of patients associated with these types of symptoms.

Furthermore, the data sources for this study (the Veradigm Practice Fusion and Next Gen EHR databases) were used by primary care outpatient practices. Accordingly, the data may have over-represented persons with milder schizophrenia and less negative symptoms compared to community mental health centers having a higher density of persons with more severe schizophrenia and possibly more negative symptoms. This raised the question of whether the presumed missed information using the NLP approach was due to (1) shortcomings in the typical clinical exam for these patients, and specifically the absence of a systematic approach to clinically collect and document data on negative symptoms, impacting the frequency and number of available clinical notes; (2) the data source; (3) the NLP methodology itself; or (4) a combination of these factors.

Finally, the study sample was not intended to be a random representative sample of US patients but instead reflected the characteristics of healthcare providers who subscribed to the EHR platform. Therefore, patterns observed in these patients may not have accurately portrayed all schizophrenia patients in the United States. The most predominant negative symptoms reported in this study, blunted/flat affect (reduced range of emotions) and avolition (reduced goal-directed activity due to decreased motivation), were different than what Bobes et al. (2010) found in patients in Spain, where social and emotional withdrawal and poor rapport were the most frequently reported symptoms.^32^ Patients included in this study were primarily located in the South, Southwest, and Northeast of the United States. Additional large, geographically diverse studies may help to further understand the prevalence and characteristics of negative symptoms in people living with schizophrenia in the United States.

This study linking EHR data to claims data and using NLP is a novel approach to identifying negative symptoms in patients with schizophrenia in the United States and understanding the characteristics of patients suffering from these symptoms. These data add to the limited published literature on negative symptoms in patients with schizophrenia in the United States. They inform the need for future treatment strategies and healthcare policies for improved recognition and management of negative symptoms among patients with schizophrenia. The low documented identification of negative symptoms observed in this study highlights the need for both patients and providers to recognize and document this important domain of schizophrenia. A standardized and consistently used assessment to identify and document negative symptoms may result in better recognition and management of this domain for patients with schizophrenia. Low utilization of psychosocial interventions along with increased costs and healthcare utilization observed in this study emphasize the need for better management and treatment options for patients with schizophrenia.

## Supplementary Material

sbaf073_suppl_Supplementary_Table_S1

sbaf073_suppl_Supplementary_Table_S2

sbaf073_suppl_Supplementary_Table_S3

sbaf073_suppl_Supplementary_Table_S4

sbaf073_suppl_Supplementary_Table_S5
