# Supplementary material for: Economic Burden Associated with Negative Symptoms Identified Through Natural Language Processing Among Patients with Schizophrenia in the United States
Source: Schizophr Bull. 2025 Jun 3;52(2):sbaf073. doi: 10.1093/schbul/sbaf073 (PMC12996878; doi:10.1093/schbul/sbaf073)
Supplement: sbaf073_suppl_Supplementary_Table_S1 [file sbaf073_suppl_supplementary_table_s1.docx]

Supplementary Table S1. Keywords and Phrases Identified by NLP

| Negative Symptoms: Keywords and phrases of interest | Category |
| --- | --- |
| (short\|brief\|monosyllable) (comments?\|answers?) | alogia |
| 1-2 word responses? | alogia |
| uses few words | alogia |
| (no response\|doesn’t speak) | alogia |
| poor responsiveness | alogia |
| not spontaneous | alogia |
| delayed speech | alogia |
| poverty of speech | alogia |
| decreased verbal output | alogia |
| reduced speech productivity | alogia |
| minimal responses | alogia |
| brief and empty replies | alogia |
| lack of spontaneous speech | alogia |
| paucity of language content | alogia |
| reduced fluency | alogia |
| diminished verbal expression | alogia |
| apathetic speech | alogia |
| restricted speech output | alogia |
| (empty\|vague) communication | alogia |
| impoverished verbal communication | alogia |
| difficulty (initiating\|sustaining) conversation | alogia |
| limited word usage | alogia |
| verbal underactivity | alogia |
| sparse speech content | alogia |
| decreased verbosity | alogia |
| minimal elaboration in conversation | alogia |
| impaired expressive language | alogia |
| (uninterested\|unenthusiastic) about (hobbies\|personal interests\|activities) | anhedonia |
| little involvement in (hobbies\|activities) | anhedonia |
| (no\|few) leisure activities | anhedonia |
| (no\|limited) anticipation of future activities | anhedonia |
| does not look forward to (events\|activities) | anhedonia |
| loss of interest | anhedonia |
| decreased pleasure in activities | anhedonia |
| inability to experience joy | anhedonia |
| diminished enjoyment | anhedonia |
| lack of enthusiasm | anhedonia |
| reduced engagement in previously enjoyable activities | anhedonia |
| emotionally flat response to pleasurable stimuli | anhedonia |
| absence of pleasure | anhedonia |
| decreased motivation to engage in rewarding activities | anhedonia |
| reduced interest in (hobbies\|recreation) | anhedonia |
| altered responsiveness to positive events | anhedonia |
| reduced anticipation of pleasure | anhedonia |
| inability to derive satisfaction from experiences | anhedonia |
| loss of (excitement\|zest) for life | anhedonia |
| diminished sense of enjoyment | anhedonia |
| lack of interest in (social interactions\|relationships) | anhedonia |
| impaired ability to experience (pleasure\|reward) | anhedonia |
| reduced reactivity to pleasurable stimuli | anhedonia |
| (apathetic\|indifferent) attitude towards pleasurable experiences | anhedonia |
| loss of motivation to pursue previously rewarding goals | anhedonia |
| (no\|few\|limited) friends | asociality |
| poor relationships | asociality |
| (no\|limited) interest in others | asociality |
| prefers isolation | asociality |
| socially inactive | asociality |
| (reduced\|suppressed) (interest\|involvement) in intimate relationships | asociality |
| social withdrawal | asociality |
| lack of interest in social interactions | asociality |
| decreased social engagement | asociality |
| isolation from others | asociality |
| social disengagement | asociality |
| avoidance of social situations | asociality |
| reduced social interaction | asociality |
| diminished social participation | asociality |
| apathy towards socializing | asociality |
| minimal social contact | asociality |
| impaired social functioning | asociality |
| limited social connections | asociality |
| decreased desire for companionship | asociality |
| withdrawal from social networks | asociality |
| absence of socializing behaviors | asociality |
| reduced interest in maintaining relationships | asociality |
| social detachment | asociality |
| loss of interest in group activities | asociality |
| impaired social reciprocity | asociality |
| social apathy | asociality |
| (no\|limited) (motivation\|initiative) | avolition |
| (no\|low\|reduced) interest in (goals\|work\|school) | avolition |
| poor (grooming\|hygiene) | avolition |
| Withdrawn | avolition |
| uninvolved in (work\|school) | avolition |
| lack of concern | avolition |
| reduced motivation | avolition |
| lack of goal-directed behavior | avolition |
| diminished drive | avolition |
| loss of interest in activities | avolition |
| decreased initiative | avolition |
| apathy towards daily tasks | avolition |
| difficulty initiating tasks | avolition |
| reduced self-initiated activity | avolition |
| passiveness\|passivity | avolition |
| loss of motivation to complete tasks | avolition |
| impaired goal-setting ability | avolition |
| lack of follow-through on plans | avolition |
| reduced productivity | avolition |
| absence of purposeful action | avolition |
| decreased effort in activities of daily living | avolition |
| loss of interest in hobbies or previously enjoyed activities | avolition |
| reduced engagement in (work\|school)( related tasks) | avolition |
| decreased goal-directed behavior | avolition |
| reduced ability to (prioritize\|plan) | avolition |
| diminished self-motivation | avolition |
| loss of drive | avolition |
| unexpressive or limited expressiveness | blunted affect |
| (no\|limited) facial expression | blunted affect |
| limited gestures | blunted affect |
| (inhibited\|flat) (vocal tone\|intonation) | blunted affect |
| diminished (facial\|vocal) expressions | blunted affect |
| poor eye contact | blunted affect |
| reduced emotional expression | blunted affect |
| flat affect | blunted affect |
| lack of emotional responsiveness | blunted affect |
| restricted range of affect | blunted affect |
| decreased facial expressiveness | blunted affect |
| absence of emotional reactivity | blunted affect |
| diminished emotional display | blunted affect |
| impaired emotional modulation | blunted affect |
| reduced intensity of emotions | blunted affect |
| apathetic facial expression | blunted affect |
| emotionally unresponsive | blunted affect |
| decreased nonverbal cues | blunted affect |
| limited emotional gestures | blunted affect |
| lack of congruence between verbal and nonverbal expressions | blunted affect |
| emotionally unexpressive | blunted affect |
| reduced variability in affective responses | blunted affect |
| absence of emotional fluctuations | blunted affect |
| blunted emotional reactivity | blunted affect |
| impoverished affective expressiveness | blunted affect |
| dull emotional response | blunted affect |
| (deficit of diminished) prosody | blunted affect |
