# Supplementary material for: Economic Burden Associated with Negative Symptoms Identified Through Natural Language Processing Among Patients with Schizophrenia in the United States
Source: Schizophr Bull. 2025 Jun 3;52(2):sbaf073. doi: 10.1093/schbul/sbaf073 (PMC12996878; doi:10.1093/schbul/sbaf073)
Supplement: sbaf073_suppl_Supplementary_Table_S2 [file sbaf073_suppl_supplementary_table_s2.docx]

**Supplementary Table S2. Confusion Matrix with Performance Metrics of the Python NLP Analysis Conducted with a Random Sample (n = 250 Patients)**

| Negative Symptom | True Positive | False Positive | False Negative | True Negative | Precision (%) | Recall (%) | F1 Score |
| --- | --- | --- | --- | --- | --- | --- | --- |
| Alogia | 14 | 0 | 5 | 231 | 100 | 0.74 | 0.85 |
| Anhedonia | 12 | 0 | 6 | 232 | 100 | 0.67 | 0.80 |
| Asociality | 6 | 0 | 1 | 243 | 100 | 0.86 | 0.92 |
| Avolition | 27 | 1 | 6 | 216 | 0.96 | 0.82 | 0.89 |
| Blunted Affect | 32 | 1 | 0 | 217 | 0.97 | 100 | 0.99 |
