# Supplementary material for: Economic Burden Associated with Negative Symptoms Identified Through Natural Language Processing Among Patients with Schizophrenia in the United States
Source: Schizophr Bull. 2025 Jun 3;52(2):sbaf073. doi: 10.1093/schbul/sbaf073 (PMC12996878; doi:10.1093/schbul/sbaf073)
Supplement: sbaf073_suppl_Supplementary_Table_S3 [file sbaf073_suppl_supplementary_table_s3.docx]

Supplementary Table S3. Study Population

| **Selection criteria** | **N (%)** |
| --- | --- |
| Patients with a diagnosis of schizophrenia at any time within the study period | 314,887 |
| Patients with two or more outpatient encounters on or after start of study period, both of which must be tied to a schizophrenia diagnosis | 191,334 (60.8%) |
| Patients with ≥12 months of activity in EHR during study period | 162,450 (84.9%) |
| Patients age ≥18 years at index date | 161,019 (99.1%) |
| Evidence of activity in the EHR at any time prior to index date | 156,530 (97.2%) |
| No evidence of non-Schizophrenia-related cognitive impairments^1^ | 100,934 (64.5%) |
| **Total EHR cohort: EHR activity ≥12 months prior to index** | **79,326 (78.6%)** |
| **Evidence of negative symptoms cohort** | **14,992 (18.9%)** |
| **Those with experiential negative symptoms** | **9,030 (60.2%)** |
| No evidence of negative symptoms cohort | 64,334 (81.1%) |
| **Total linked claims cohort: total EHR cohort with linked claims** | **11,293 (14.2%)** |
| **Evidence of negative symptoms** | **1,975 (17.5%)** |
| **Those with experiential negative symptoms** | **1,177 (59.6%)** |
| No evidence of negative symptoms | 9,318 (82.5%) |

^1^Evidence of stroke, dementia, prion disease, multiple sclerosis, or traumatic brain injury prior to index; or autism spectrum disorder, epilepsy, or intellectual disability at any time

Abbreviations: EHR, electronic health records
