# Supplementary material for: Economic Burden Associated with Negative Symptoms Identified Through Natural Language Processing Among Patients with Schizophrenia in the United States
Source: Schizophr Bull. 2025 Jun 3;52(2):sbaf073. doi: 10.1093/schbul/sbaf073 (PMC12996878; doi:10.1093/schbul/sbaf073)
Supplement: sbaf073_suppl_Supplementary_Table_S4 [file sbaf073_suppl_supplementary_table_s4.docx]

Supplementary Table S4. Annual healthcare utilization for patients with any negative symptoms and for patients with documented experiential negative symptoms in the linked claims cohort compared to patients without documented negative symptoms

|  | Documented Negative Symptoms | P-value | Documented Experiential Negative Symptoms | P-value |
| --- | --- | --- | --- | --- |
|  | N = 1,975 |  | N = 1,177 |  |
| Annual Healthcare resource utilization PPPY (Mean) |  |  |  |  |
| Number of outpatient visits | 46.9 | <0.001 | 46.8 | 0.001 |
| Total hospitalized days | 5.2 | <0.001 | 5.3 | 0.015 |
| Number of hospitalizations | 0.4 | 0.001 | 0.5 | 0.001 |
| Number of emergency department visits | 1.6 | 0.112 | 1.44 | 0.448 |
| Number of all-cause claims^1^ | 86.4 | <0.001 | 85.2 | 0.006 |
| Number of pharmacy claims | 37.2 | 0.137 | 37.2 | 0.675 |
