# Supplementary material for: Economic Burden Associated with Negative Symptoms Identified Through Natural Language Processing Among Patients with Schizophrenia in the United States
Source: Schizophr Bull. 2025 Jun 3;52(2):sbaf073. doi: 10.1093/schbul/sbaf073 (PMC12996878; doi:10.1093/schbul/sbaf073)
Supplement: sbaf073_suppl_Supplementary_Table_S5 [file sbaf073_suppl_supplementary_table_s5.docx]

Supplementary Table S5. Annual healthcare costs for patients with any negative symptoms and for patients with experiential negative symptoms in the linked claims cohort compared to patients without evidence of negative symptoms

|  | Documented Negative Symptoms | P-value | Documented Experiential Negative Symptoms | P-value |
| --- | --- | --- | --- | --- |
|  | N = 1,975 |  | N = 1,177 |  |
| Annual Healthcare costs, PPPY (Mean) |  |  |  |  |
| Inpatient costs | $7,354.20 | <0.001 | $6,279.36 | 0.007 |
| Emergency department visit costs | $1,772.04 | 0.397 | $1,621.92 | 0.797 |
| Outpatient costs | $16,765.60 | <0.005 | $16,928.76 | 0.061 |
| Pharmacy costs | $5,464.68 | 0.052 | $5,593.92 | 0.054 |
| All-cause healthcare related costs | $31,356.50 | <0.001 | $30,423.84 | 0.005 |
| Schizophrenia-related healthcare costs | $23,428.40 | <0.001 | $15,060.60 | <0.001 |
